# An assembled molecular signaling map of interleukin-24: a resource to decipher its multifunctional immunoregulatory role in pathophysiological conditions

**DOI:** 10.3389/fimmu.2025.1608101

**Published:** 2025-06-30

**Authors:** Anjana Aravind, Vineetha Shaji, Mukhtar Ahmed, Shobha Dagamajalu, Manavalan Vijayakumar, Thottethodi Subrahmanya Keshava Prasad, Rajesh Raju

**Affiliations:** ^1^ Center for Systems Biology and Molecular Medicine, Yenepoya Research Centre, Yenepoya (Deemed to be University), Mangalore, Karnataka, India; ^2^ Department of Zoology, College of Science, King Saud University, Riyadh, Saudi Arabia; ^3^ Department of Surgical Oncology, Yenepoya Medical College, Yenepoya (Deemed to be University), Mangalore, Karnataka, India; ^4^ Center for Integrative Omics Data Science (CIODS), Yenepoya (Deemed to be University), Mangalore, Karnataka, India

**Keywords:** tumor suppressor cytokine, immune cells, therapeutic agent, immuno-stimulation, signaling pathway

## Abstract

**Introduction:**

Interleukin-24 (IL-24) is a cytokine belonging to the IL-10 family with immunoregulatory properties. It is known to induce cellular responses in various disease conditions, including inflammatory, infectious diseases, and cancer, in a receptor-dependent and independent manner. IL-24 is induced by both immune and non-immune cells and acts on a vast array of target cells to regulate various biological processes. The antitumor and immunoregulatory properties of IL-24 support its application as a therapeutic agent in a vast array of pathological conditions. Characterizing the molecular events associated with its immunomodulatory properties is essential for evaluating its utility in cancer immunotherapy.

**Methods:**

The data related to signaling events induced by IL-24 were then manually annotated and cataloged from the literature pertaining to IL-24 signaling. The cataloged molecular events were then manually drawn based on the topology as a signaling pathway map of IL-24.

**Results:**

Based on the data mined from published literature, we assembled IL-24-regulated signaling events linked to its immunoregulatory and anti-tumor activities. The receptor-ligand interaction of IL-24 regulates multiple downstream signaling cascades, leading to antitumor activities alongside inducing inflammatory reactions and anti-infectious processes. We provide a signaling pathway map of IL-24 consisting of 433 mRNAs/proteins and their 613 signaling events.

**Discussion:**

We believe this map would serve as a knowledge base for reference and one-go visualization of the signaling events identified to be induced by IL-24. Moreover, this map is made available (https://ciods.in/il24/) for the researchers to explore the role of IL-24 in various pathophysiological conditions through the gene set enrichment of transcriptomics or proteomics datasets from various disease conditions.

## Graphical Abstract

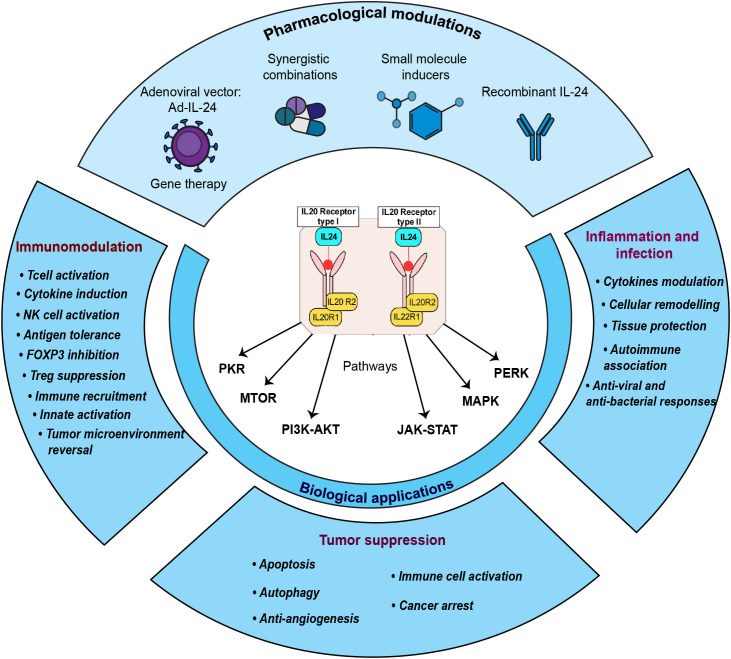

## 1 Introduction

Interleukin 24 (IL-24) is a multifunctional cytokine belonging to the IL-10 subfamily with immunomodulatory functions (1–3). It is also known as melanoma differentiation-associated gene-7 (MDA-7), as it was first isolated from growth-arrested and terminally differentiated human melanoma cells using subtractive hybridization (4). It is also known as ST16 (suppressor of tumorigenicity-16) and was renamed IL-24 after the discovery of cell surface receptors of MDA-7 (5–9). IL-24, with its unique cytokine properties, has dual functions as a pro- and anti-inflammatory agent and as a tumor suppressor cytokine (10, 11). It exhibits the ability to selectively induce cell death in various cancer cells (2, 10, 11). It is a pleiotropic cytokine studied in several inflammatory and cancer conditions, known for its characteristics to modulate immune cell activity with diverse functions depending on the target cell type and disease state (10). This secretory cytokine consists of 206 amino acid residues, with 51 amino acids serving as a signal peptide (12). The human IL-24 gene is located on chromosome 1, within a 195-kb cytokine cluster named the “IL-10 family gene cluster,” comprising four genes, IL-10, IL-19, IL-20, and IL-24. IL-24 shares a high sequence homology at the amino acid level across the species (9).

IL-24 is produced by immune cells of both lymphoid and myeloid lineages when activated with certain lipopolysaccharides or specific cytokines and non-immune cells, including melanocytes (1, 12). It originates mainly from activated monocytes and T helper 2 cells. IL-24 functions as a classical cytokine, exerting its effects through its cell-surface heterodimeric receptor complex (IL-20R1 and IL-20R2; IL-22R1 and IL-20R2). IL-20R1 and IL-22R1 pair with the IL-20R2 chain to form a heterodimeric receptor complex, thereby inducing cell signaling events (13, 14). The IL20R1 and IL22R1 expression levels determine the quantity of these two receptor complexes. The expression of the three subunits was consistent in the majority of the tissues and cellular conditions (6, 15). This heterodimeric receptor complex is a part of the IL-10 receptor family and is shared among cytokines within this family, which displays a 20-30% sequence homology (6–8). The utilization of shared receptors is a common mechanism within the cytokine family, leading to the cross-talk and precise regulation of cellular response in the immune system (9). Moreover, IL-24 may interact differently with type I and type II receptor heterodimers, exerting non-redundant and unique biological activities (15). In addition to receptor-ligand interaction-induced signaling events, IL-24 initiates multiple signaling events intracellularly, independent of its non-cognate receptors (16, 17).

IL-24 is a versatile cytokine that can regulate anti-tumor activity, immune response, tissue homeostasis, and host defense mechanisms (3, 10). This multifunctional protein affects broad types of cancer and has a crucial role in regulating cell proliferation, survival, tumor suppression, differentiation, and apoptosis-inducing properties (18, 19). It is well-known for its multi-pronged indirect anti-tumor activities, including the induction of the release of other cytokines, resulting in immunomodulatory activities (1, 20, 21). It can also regulate the proliferation and activation of CD4+ and CD8+ T cell populations (22, 23). Due to its remarkable tumor-suppressing ability, IL-24 shows greater potential for advanced targeted cancer therapy. Recent reports have demonstrated the immunotherapeutic potential of IL-24, with its immunomodulatory activity combined with the apoptotic and anti-angiogenic properties, strengthening its utility in cancer immunotherapy (24–28). A Phase I clinical trial using an adenovirus vector expressing IL-24 (Ad-IL-24) has documented the T cell activation and cytokine release for the eradication of tumor cells (28–36). The strategic utilization of IL-24 in cancer therapy leads to a promising method of precision medicine, navigating toward an effective approach in cancer treatment.

Despite the importance of IL-24-mediated signaling, comprehensive information on IL-24-mediated signaling events is currently limited. The KEGG and Reactome pathway databases only offer a general overview of the signaling modules regulated by IL-24. The potential roles of IL-24 as a cytokine in the form of a comprehensive signaling pathway map will encourage further investigation into IL-24-mediated cellular events. This may lead to unraveling specific roles of individual molecules and modules in various pathophysiological conditions, including cancer, inflammatory disorders, and other diseases. Previously, we compiled the signaling events of several other cytokines, including CCL18, CCL19, IL-11, IL-17, IL-19, and 1L-20, through the documentation of their cell-specific functional roles attributed to diverse experimental conditions (37–41). Similarly, the study-centric molecular reactions of IL-24 were assembled, adding them to the compendium of cytokine signaling pathway maps for gene set enrichment analyses.

## 2 Methods

### 2.1 Data curation of IL-24 signaling events

To create a comprehensive IL-24 signaling pathway map, a literature survey was carried out on IL-24 signaling. The search terms used were “IL-24 AND Signaling,” and the articles were retrieved from PubMed. The abstract of research articles describing the signaling events induced by IL-24 was manually screened to select the relevant ones. The experimental datasets were limited to the signal transduction events induced by IL-24, excluding the knock-out and co-expression studies. The curated datasets encompass studies pertaining to diseases such as cancer, infectious, and inflammatory conditions.

### 2.2 Functional annotation and assembly of the signaling pathway data

**Figure 1:**
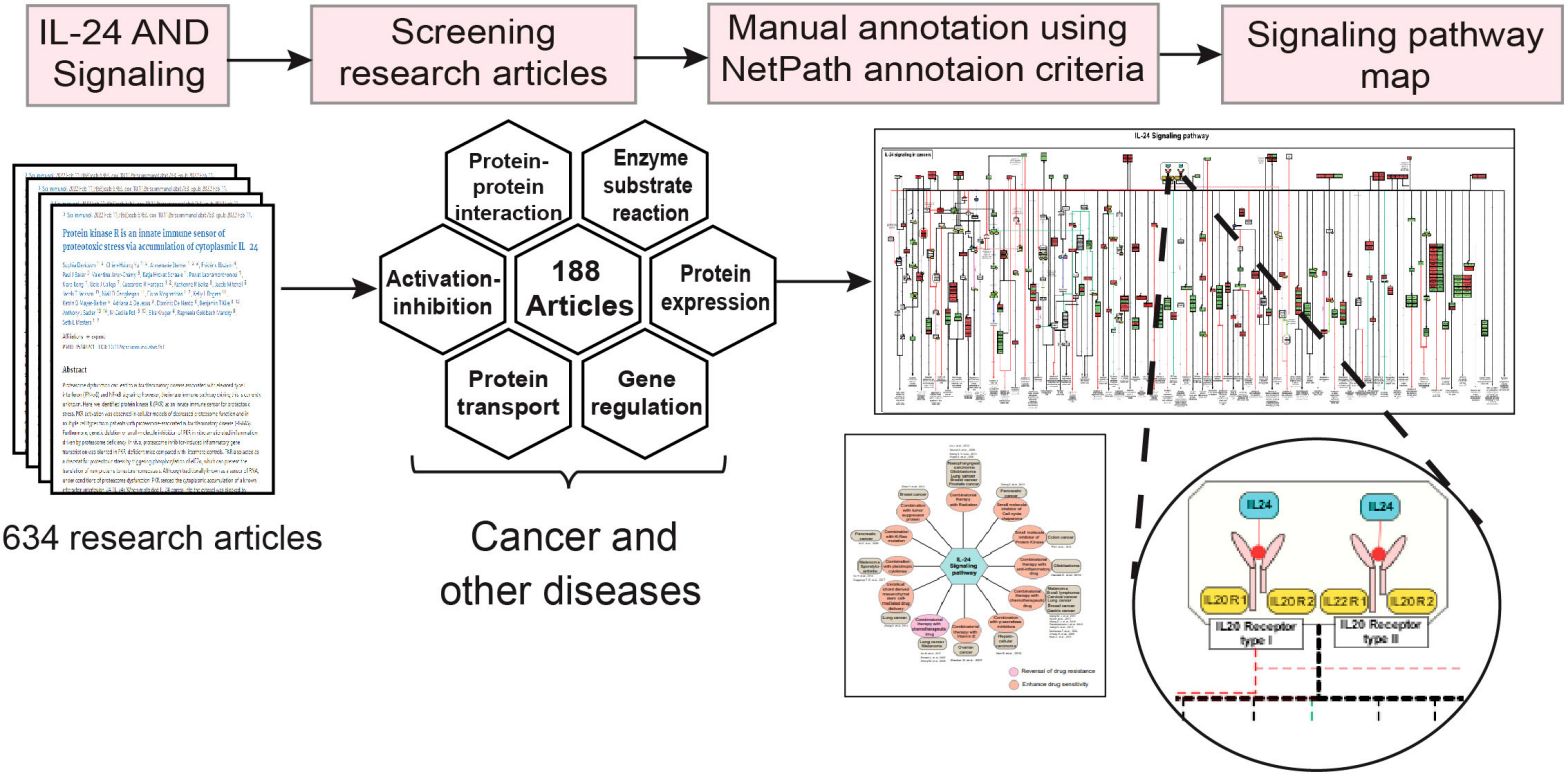
A detailed workflow outlining the methodology for the IL-24 signaling pathway data curation, annotation, and map development. The workflow depicts the sequential steps used to develop the IL-24 signaling pathway map. The literature related to IL-24 signaling in cancer, infectious, and inflammatory disease conditions was manually screened, followed by the curation and annotation of the signaling events according to the NetPath criteria. Further, with the annotated data, a detailed signaling pathway map was developed.

The data related to signaling events induced by IL-24 were then manually annotated and cataloged according to the PathBuilder criteria (**Figure 1**) (42). IL-24-induced molecular events such as protein-protein interactions, protein expression, gene regulation events, activation/inhibition events, enzyme catalysis/post-translational modifications (PTM), molecular association, and protein translocation were curated from the research articles (43). The protein and gene expression events were cataloged for overexpressed (fold change >1.3, p-value <0.05) and downregulated (fold change <0.76, p-value <0.05) molecules, as devised in each of the studies. All possible PTM events, including acetylation, ubiquitination, denitrosylation, N-glycosylation, phosphorylation, and dephosphorylation, with their site-specific information, were annotated within the pathway map and are delineated with different colors. Further, the translocation events were categorized based on the transportation of proteins within the cellular organelles comprising the nucleus, plasma membrane, cytoplasm, mitochondria, and Golgi complex. The proteins and genes were mapped to their corresponding Entrez Gene ID to annotate the data in a standardized format. Besides the proteins, the involvement of small molecules or second messengers such as reactive oxygen species (ROS), Ca^2+^, and cAMP was also noted. Individual study-centric signaling events and their related disease conditions and cell type-specific reaction events were documented. The data also included the type of experiments performed to detect and validate the expression of downstream molecules of IL-24 ligand-receptor interaction and the cell line/tissue used. The data on the synergistic activities of IL-24 were curated using the same data annotation strategy and included information on the impact of drugs and other molecules of therapeutic potential.

### 2.3 Development of the IL-24 network map

The annotated molecules, reactions, and disease-specific biological processes were manually drawn based on the topology as a signaling pathway map of IL-24 using the PathVisio (version 3) tool (44). The topology that represents the spatial order of the molecules was devised based on the directionality initiated by the IL-24 and its receptor interaction, as determined by studies using inhibitors or the established cascade of reactions.

### 2.4 Omics data integration and PPI mapping

The protein-protein interaction network analysis was performed with IL-24, its receptors, and downstream targets. The analysis was carried out using the tool STRING database with a confidence score of >0.7 (45). To enrich the functional clusters, the network was imported into the Cytoscape tool v 3.10.2, and the algorithm Molecular Complex Detection (MCODE) was applied (46, 47). MCODE identifies the clusters based on their local neighborhood density and expands from highly connected hubs. Additionally, publicly available omics datasets were analyzed to provide supporting evidence for the pathway map. The PBMC transcriptomic data were retrieved from the NCBI GEO DataSets and analyzed using GEO2r to assess the expression of IL-24 across various disease conditions. Similarly, differentially expressed proteins were obtained from the publicly available proteomic dataset, and the pathway enrichment analysis was performed using the Reactome database (48).

## 3 Results and discussion

To develop the IL-24 signaling pathway map, a total of 697 articles were screened in PubMed using the search criteria described in the methods section. A total of 341 molecules involved in IL-24-mediated 602 signal transduction events were manually vetted from the 191 studies. These events include 79 enzyme-substrate reactions, 15 protein-protein interactions, 76 activation/inhibition reactions, 10 protein translocation events, 262 protein expressions, and 171 gene regulation events identified from the annotations of these selected articles. Information on differentially regulated mRNA/proteins responding to IL-24 stimulation is also available. Among the 171 genes, 93 were overexpressed, and 78 were downregulated, whereas in 262 proteins, 132 were overexpressed, and 130 were downregulated. As far as we know, this is the first pathway resource that catalogs IL-24-mediated molecular reactions. The network map depicts the molecular events involved in a wide range of IL-24-induced processes, including host defense, tissue homeostasis, and immunological regulation in various physiological conditions.

**Figure 2:**
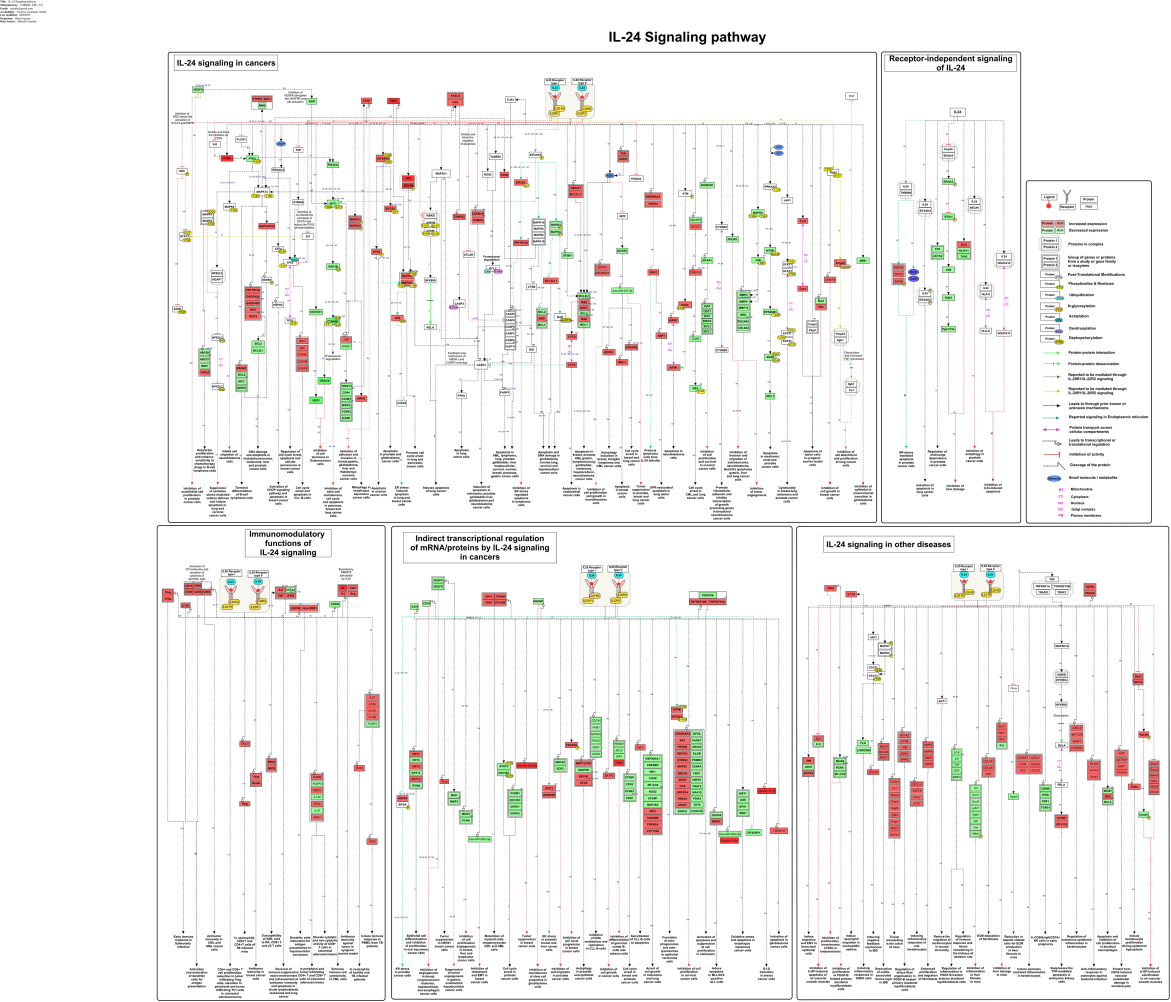
The schematic representation of IL-24-induced signaling reactions. The signaling pathway map shows molecules associated with ligand-receptor interactions and IL-24 downstream molecular events. The pathway comprises the signaling modules such as activation/inhibition, enzyme catalysis, protein/gene regulatory events, molecular association, and translocation events induced by IL-24 toward its anti-tumor and immunoregulatory properties. These signaling events are represented with a specific type of edge and are color-coded as mentioned in the pathway legend. The pathway also includes information about the site and residues of post-translational modifications. The functions/processes linked to each signaling event, as annotated from the curated study, are displayed at the bottom of the pathway.

The study-centric molecular reactions of IL-24 in various pathological conditions comprising cancer, infection, and other diseases were manually annotated, assembled, and represented as a comprehensive signaling pathway map (**Figure 2**). The detailed annotated data that were analyzed for building the IL-24 pathway map are provided in the **Supplementary File S1**. As supported by the assembled data, the functional role of IL-24 is facilitated either through the heterodimeric receptor complexes (IL-20R1 and IL-20R2; IL-22R1 and IL-20R2) or intracellularly, independent of the receptors. The receptor-mediated functional role of IL-24 was observed to be initiated through both of these receptor complexes in the majority of the investigated studies. The IL-24-IL-20R1/IL-20R2 and IL-24-IL-22R1/IL-20R2 ligand-receptor interactions are reported to mediate the cellular responses through multiple signaling modules encompassing JAK-STAT, PI3K-AKT, mTOR, CHOP, MAPK, PERK, and PKR. This heterodimeric receptor complex is also shared within the other IL-10 cytokine family members, allowing an overlapping core signaling mechanism, particularly involving the IL-22R1 and IL-20R2 and the JAK-STAT signaling pathway in epithelial cells, keratinocytes, and immune cells (49). Even though IL-24 is structurally related to other IL-10 family members and shares some signaling machinery, it is unique in its anti-tumor properties, endoplasmic reticulum (ER) stress, and reactive oxygen species (ROS) production that induce selective toxicity in cancer cells (11, 50). These intricate signaling networks contribute to the diverse physiological effects elicited by IL-24 in various biological contexts.

### 3.1 Deciphering the role of IL-24 as a tumor suppressor cytokine

The distinctive functions of IL-24 in cancer include tumor-specific killing through an integrated effect of apoptosis, leading to a potent “bystander” anti-cancer activity, inhibition of cell proliferation, migration, and angiogenesis (18). This cytotoxic activity could cause either growth arrest or apoptosis through various cellular mechanisms solely in cancer cells at the supraphysiological levels of IL-24. Meanwhile, IL-24, at its natural physiological level, is recognized to function regularly to maintain its cellular processes and immune responses. A potential explanation for this selectively induced apoptosis is the intrinsic biochemical distinctions between normal and cancer cells. This encompasses factors such as high ROS production, altered metabolism, reprogrammed signaling pathways, and oxidative and endoplasmic reticulum (ER) stress (51). The bystander activity of IL-24 induces anti-cancer effects on IL-24 target cells and influences the neighboring untreated cells. This intriguing property makes IL-24 a promising candidate for cancer therapy as it amplifies its therapeutic potential beyond the directly treated cells. The IL-24 expression in tumor cells is primarily derived from tumor-infiltrating immune cells, such as T cells and NK cells, rather than from the tumor cells themselves. Moreover, the cells transduced with IL-24 can secrete other cytokines acting on the adjacent tumor cells, exerting their apoptotic and anti-proliferative effects (11). The tumor suppressive activity of IL-24 has been widely studied in a broad range of cancers, including oral, breast, lung, cervical, and colorectal carcinoma (22, 52–55).

#### 3.1.1 Induction of apoptosis through IL-24 signaling axis

IL-24 modulates diverse apoptosis-related signaling pathways through the regulation of multiple apoptosis-effector proteins in various cancers. It promotes apoptosis through the extrinsic pathway, mediated by death receptors (56, 57). It also invokes the intrinsic pathway, driven by the expression of Ataxia Telangiectasia Mutated protein (*ATM*) and TP53. This intricate network stimulates pro-apoptotic and inhibits anti-apoptotic molecules, culminating in the activation of caspases, orchestrating the subsequent cell death across diverse cancer types (58–60). Additionally, it initiates apoptosis through oxidative and ER stress by triggering the release of ROS, activation of PKR (*EIF2AK2*) and multiple MAPKs, followed by the activation of ER stress markers, DNA damage-inducing proteins, and BiP (*HSPA4*) (51, 56, 61–63). Despite the activation of apoptosis, IL-24 is also known to induce autophagy-associated cell death by the regulation of miR-221 and Beclin 1 (*BECN1*) axis and direct activation of autophagy-related gene 5 (*ATG5*) (64, 65). Besides, IL-24 is well-known for the phosphorylation, activation, and nuclear translocation of the tumor suppressor protein TP53, resulting in cell proliferation arrest (66). Altogether, these combined actions culminate in the initiation of apoptosis within several cancer types.

#### 3.1.2 Diverse mechanisms of IL-24-mediated anti-tumor activity

IL-24 exhibits potent anti-tumor activity, including anti-angiogenesis by suppressing endothelial cell markers such as vascular endothelial growth factors and *CD34*. It induces cell cycle arrest (G2-M phase) by modulating cell cycle proteins such as CDKs, cyclins, and cell division cycle proteins. Further, it inhibits cancer cell adhesion and invasion by downregulating matrix metalloproteinases (MMPs) and adhesion molecules (67–70). IL-24 has also proven to be an anti-proliferative molecule, substantiated by its ability to reduce the expression of proliferation markers, Marker of Proliferation Ki-67 (*MKI67)*, and Proliferating Cell Nuclear Antigen *(PCNA*) (71, 72). In addition, IL-24 has a significant role in impeding cancer stem cell maintenance and epithelial-to-mesenchymal transition (EMT), reversing drug resistance, and inducing cellular senescence in cancer cells. Additionally, it involves the inhibition of both Notch and the Wnt/β-catenin signaling pathways with the subsequent proteasomal degradation of β-catenin (*CTNNB1*), and the ubiquitination and degradation of *BCL2* (52, 71, 73–78).

### 3.2 Synergistic activities of IL-24

**Figure 3:**
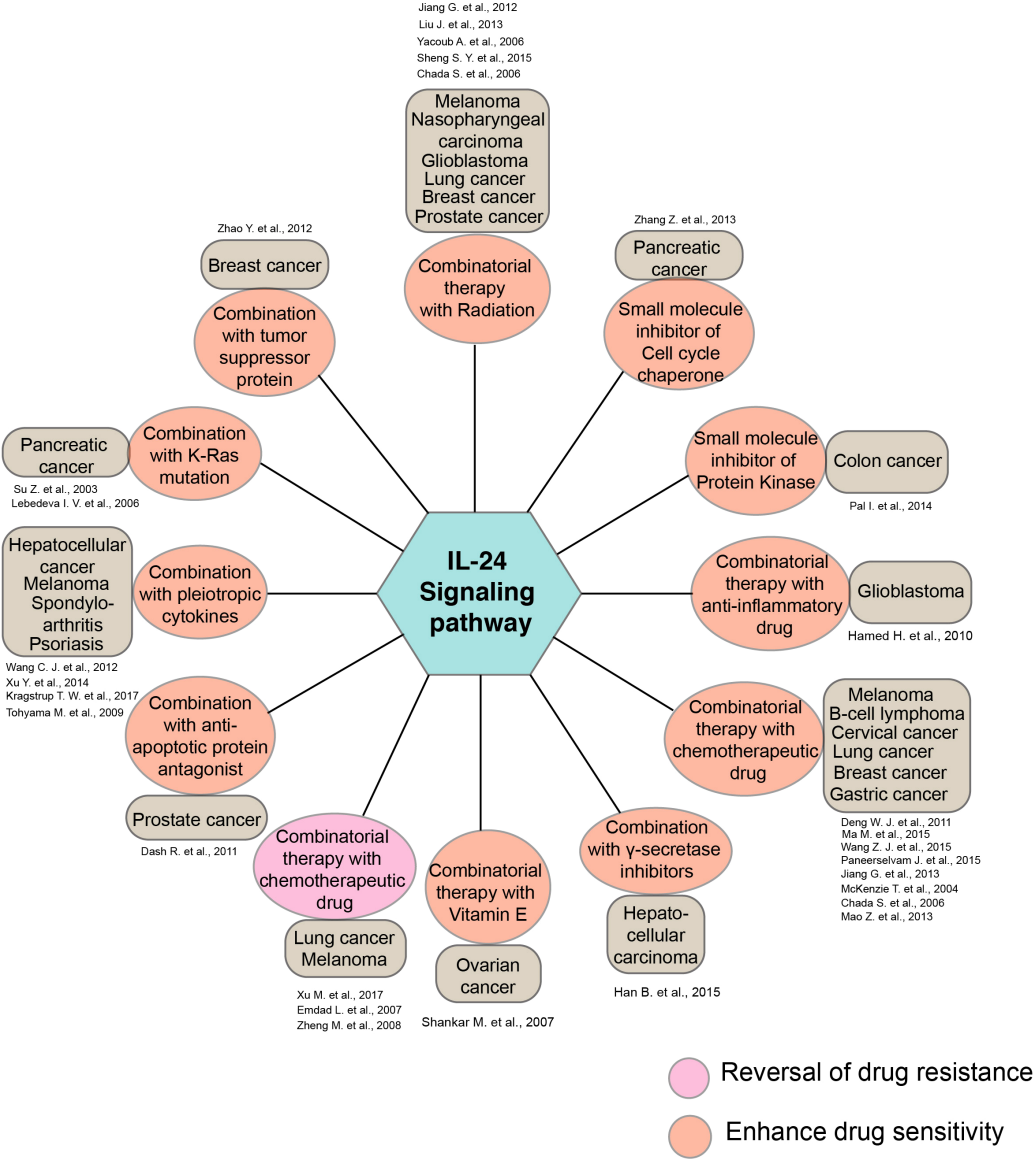
Diagrammatic illustration of the synergistic activity of IL-24 along with the various drugs and therapeutic agents. The diagram summarizes the synergistic action of IL-24 and other pharmacological agents in various disease conditions. Key drugs and the cancer conditions indicated in the study are represented along with the amplified therapeutic responses.

Beyond the intrinsic property of IL-24 as a cytotoxic and tumor-suppressant agent, numerous studies have investigated the utility of IL-24 with other drugs as a combinatorial drug administration approach in various diseases (**Figure 3**). Research findings have demonstrated that combining multiple chemotherapeutic agents and radiation with IL-24 in cancer treatment has amplified the effectiveness of chemotherapy and radiotherapy in numerous cancers (79–81). Also, studies have shown that IL-24 promotes apoptosis in various cancer types, boosting its anticancer activity when used in conjunction with some anti-inflammatory drugs, pleiotropic cytokines, including IL-20, IFNG, IL-6, vitamin E, gamma-secretase, and other small-molecule inhibitors (82–84).

### 3.3 IL-24 signaling in inflammation and infectious diseases

IL-24 can be a potent therapeutic agent against viral and bacterial infections like influenza, Human immunodeficiency virus (HIV), *Mycobacterium*, and *Salmonella* (85). As well, in inflammatory conditions such as endometriosis, inflammatory bowel diseases (IBD), asthma, and psoriasis, IL-24 has been observed to modulate inflammation-associated events (86–90). Higher expression levels of IL-24 have always been associated with chronic inflammation and autoimmune disorders such as psoriasis, arthritis, vascular permeability, and IBD (91). In chronic inflammatory conditions such as asthma, IL-24 contributes to inflammation by promoting EMT of alveolar cells and neutrophils (89, 90). While in pathogenic infections, IL-24 induces an immune response through the secretion of cytokines such as IL-12, IL-23, IL-27, and IFNG (20, 92). IL-24 induces cytokine secretion by the activation of SOCS proteins through the JAK-STAT pathway. The SOCS proteins are known to play an important role in IL-24 signaling cascades for various inflammatory and infectious conditions (14).

The role of IL-24 in inflammatory diseases is context-specific, where it has been implicated in promoting inflammation in certain pathophysiological conditions. Similar to the case of cancer, IL-24 functions to reduce the proliferation and invasiveness of endometrial squamous cells (ESCs) in endometriosis, marked by the downregulation of *MKI67* and *PCNA*. In addition, IL-24 also suppresses the macrophage-induced high viability and invasiveness of ESCs (88). Whereas, in IBD, recent studies have found that peripheral blood mononuclear cells (PBMCs) and epithelial cells secrete higher amounts of IL-24, resulting in the activation of extracellular matrix (ECM) and mucin proteins (collagens, fibronectins) contributing to the remodeling of the mucous layer in IBD (93, 94). In other inflammatory conditions, such as psoriasis, IL-24 is highly stimulated and induces the expression of the inflammatory mediators, including Psoriasin, chemokine ligands (87).

Furthermore, studies have proven that IL-24 can act as a protective agent and reverse the molecular events caused by the injuries induced by specific agents, such as H_2_O_2_ and β-glycerophosphate (95, 96). Similarly, in liver fibrosis, IL-24 functions to protect the liver from inflammation and injury. IL-24 protects the liver cells through the activation of anti-oxidant proteins and the reduction of ER stress-related DNA-damaging proteins, pro-apoptotic and pro-inflammatory proteins (97, 98). Also, its potential as a therapeutic agent in central nervous system autoimmunity has been recently investigated, revealing that IL-24 suppresses ocular inflammation by markedly inhibiting the Th1 and Th17 cells differentiation and cytokine secretion from the ocular infiltrating pathogenic Th1 and Th17 cells (99). In inflammatory conditions, IL-24 plays a dual role in Th17 cells by promoting mitochondrial recruitment of STAT3, enhancing oxidative phosphorylation, and dampening the nuclear transcriptional activity of STAT3, thereby regulating the IL-10 expression and restraining the Th17-mediated immunopathology (100). Additionally, IL-24 also acts as a downstream regulator in the IL-17A-mediated signaling loop within Th17 cells, controlling the expression of other Th17-associated cytokines such as IL-17F and GM-CSF (101). Thereby, IL-24 effectively modulates the balance between inflammation and regulation in autoimmune diseases by orchestrating Th17 cell function and cytokine expression. Therefore, the intricate involvement of IL-24 in modulating immune responses and inflammatory processes unravels its potential as a therapeutic target in a range of inflammatory conditions.

### 3.4 IL-24 as an immunoregulatory cytokine

IL-24 is widely recognized as an immunomodulatory cytokine with the property to enhance the tumor-specific immune response and disrupt the tolerance toward tumor antigens (102). Recent reports indicate that IL-24 can augment CD4+ and CD8+ T cell populations and reduce T regulatory cells within tumor-infiltrating lymphocytes (TILs), suggesting IL-24-mediated anti-tumor immune responses in colorectal adenocarcinoma (22). Also, Ma et al. (2016) have reported IL-24 mediates tumor suppression through increased percentages of CD45+CD4+ and CD45+CD8+ cells and decreased percentage of CD45+CD4+Foxp3+ cells in IL-24-treated colon cancer (102). The enhanced cytolytic activity of CD8+ T cells was marked by the IFNG, granzymes, and perforins secretion stimulated by IL-24 (22). Moreover, IL-24 markedly enhanced the mRNA expression of the transcription factor T-bet (Th1 transcriptional factor) to activate CD4+ T cells. It also inhibits the expression of a T regulatory cell activation marker, transcriptional factor Forkhead Box P3 (FOXP3), ultimately reducing the T regulatory cells in colorectal carcinoma (22, 102). Alongside, it also activates and enhances the functions of the cluster of differentiation 80 and 86 (*CD80, CD86*) proteins that are known for the transduction of secondary signals for T-cell activation. Furthermore, it also stimulates innate and adaptive immune responses in myelogenous leukemia cells through the production of ligands (*MICA* and *MICB*) for Killer Cell Lectin Like Receptor K1 (*KLRK1*) on NK and T cells (103).

Besides its intrinsic immunoregulatory functions, IL-24 can induce the secretion of other cytokines and modulate its activity in diverse pathological conditions (104, 105). IL-24 stimulates the secretion of IL-6, IL-12, IL-27, and IFNG in cancer and infectious disease conditions. Thereby, IL-24 plays a significant role in regulating the immune response against tumorigenesis by reversing the immunosuppressive tumor microenvironment and promoting anti-tumor immunity (103). Additionally, in infectious disease conditions, it stimulates the immune response through the recruitment of CD4+, CD8+ T cells, and neutrophils by the activation of T-Cell-Specific T-Box Transcription Factor (*TBX21*), leading to the secretion of *IFNG* and several other interleukins (106). This orchestrated immune activation by IL-24 plays a crucial role in combating pathogens, external antigens, and tumor cells, thereby promoting the host-defense mechanisms.

### 3.5 Therapeutic applications of IL-24

The demonstration and visualization of the mechanisms of IL-24-mediated immunoregulation facilitate and aid in developing and utilizing IL-24 therapeutic approaches. Recent reports have proven the efficacy of IL-24 in enhancing CAR-T cell therapy against cancer stem cells (CSCs) that exhibit therapeutic resistance (25). The study by Hu et al. (2021) has reported that IL-24 potentially enhances the vitality of T cells, which is a common cell resource for CAR-T products, and significantly inhibits the viability and proliferation of cancer cells in leukemia and lymphoma (26). In addition, it was found that IL24-potentiated T-cell expansion and increased tumor infiltration significantly inhibited the cancer progression. Therefore, arming T cells with IL-24 shows the capabilities of tumor eradication and T-cell expansion within the tumor microenvironment. This highlights a promising approach of cellular immunotherapy that inhibits tumor escape (27). Moreover, through the annotation of IL-24 signaling mechanisms, we identified that IL-24 has the potency to significantly suppress the expression and activity of the T regulatory cell transcription factor, FOXP3, in cancer cells and infectious disease conditions. This transcription factor is well known for activating T regulatory cells that impair the treatment response in cancer immunotherapy (107). Extensive research activities are carried out to suppress this transcription factor as a cancer therapeutic approach (108). However, the ability of IL-24 to specifically target and suppress the function of FOXP3 remains inadequately investigated. Therefore, with this signaling pathway map, we were able to showcase all possible signaling mechanisms, primarily in cancer as well as in other inflammatory and infectious diseases. IL-24 stands out as a potent therapeutic agent, with supporting evidence highlighting its potential in developing advanced and personalized cancer therapeutics.

### 3.6 Applications of the IL-24 signaling pathway map to gene expression analyses

The current pathway model serves as a tool that integrates molecular-level information derived from individual studies and does not represent a temporally orchestrated network within a single cell; rather, it represents standalone findings from each study. This applies to all pathway models available to date and is further augmented by the limitations in their effective visualization (37, 43, 109). In a way, most maps are static, considering the signaling network highly spatiotemporal and dynamic. However, the pathway model represents the collective information on the molecules and their reactions that are identified to be perturbed upon IL-24 stimulation into a computationally encoded form for incorporation into enrichment tools. These details, which are otherwise scattered across the literature, would be time-consuming for individual researchers to compile into pathway models. Additionally, the present study does not provide new *in vivo* or clinical validation. However, all the signaling events incorporated into the pathway were curated from experimentally validated studies, reflecting the available literature up to the time of publication. Even though challenged by the above limitations, the current model serves as a reference platform and database to update molecular data as more studies on IL-24 become available. We also aim to refer to notable studies in the map to ensure transparency and to support future updates as more data becomes available.

**Figure 4:**
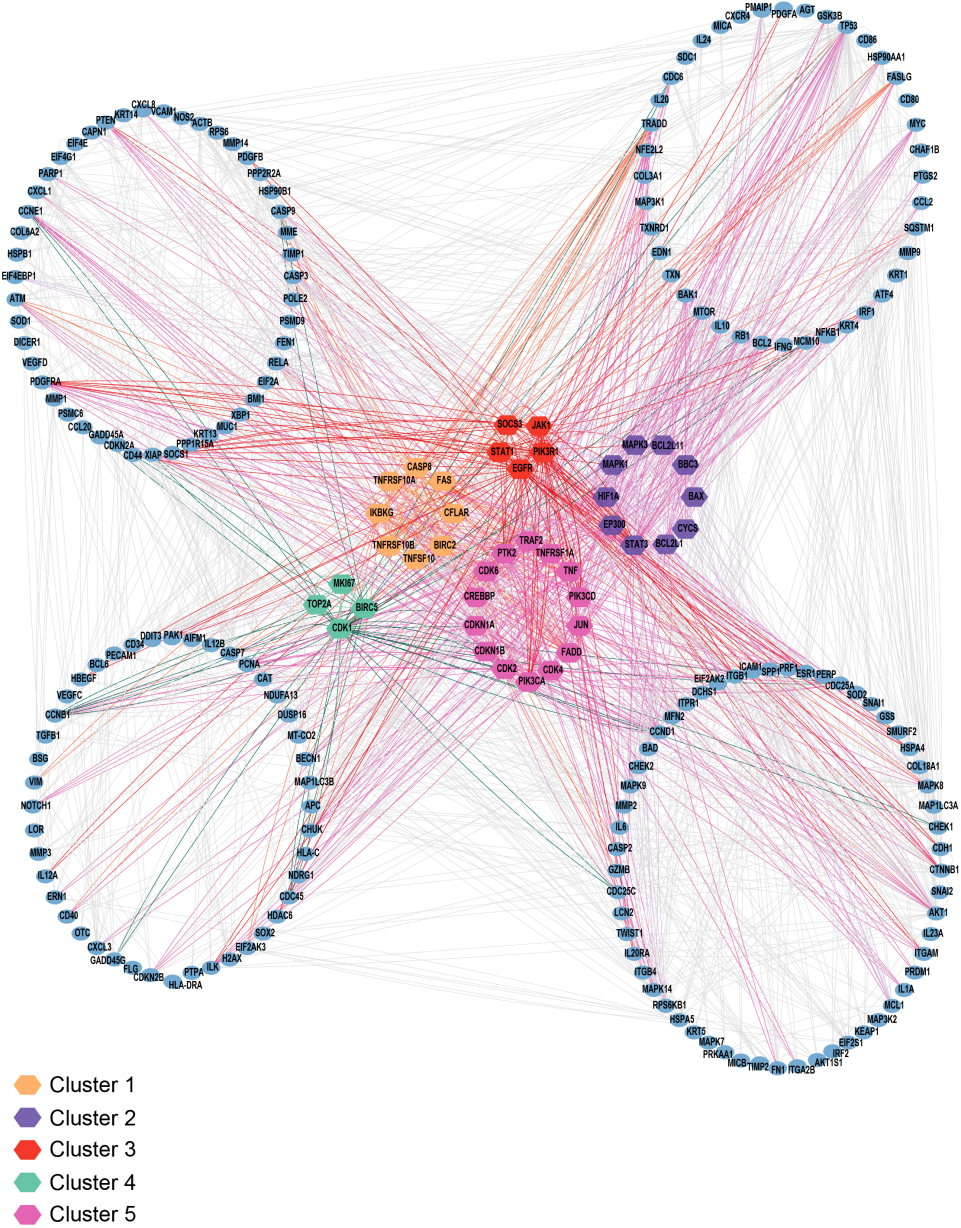
Protein-protein interaction network analysis. The figure represents the interaction network analysis of IL-24, its receptors, and downstream targets. The proteins are color-coded according to five distinct clusters based on the hub genes analysis. The hub genes are highlighted within each cluster, and the blue colored nodes depict the proteins that interact directly or indirectly with these hub genes.

Furthermore, to demonstrate the utility of the annotated signaling pathway map serving as a framework for applying gene set enrichment analysis, we mapped it into the protein-protein interaction network comprising IL-24, its receptors, and downstream targets. The analysis highlighted the various hub genes, including key signaling modules such as JAK-STAT, SOCS, PI3K, PTK2, and MAPK, together with apoptosis and cell cycle regulator proteins (**Figure 4**). This emphasizes the role of the curated signaling map in the detection of enriched signaling modules and the selection of candidate molecules for further analysis. Additionally, we looked into the publicly available transcriptomic profiles of peripheral blood mononuclear cells in cancer and infectious disease conditions. We identified three transcriptomic datasets where IL-24 expression was detected (110–112). Notably, IL-24 was consistently downregulated in cancer and inflammatory conditions, together with the suppression of anti-tumor cytokine genes within the immunosuppressive microenvironment. Whereas, in the infectious disease condition of tuberculosis, IL-24 was found to be overexpressed, indicating its anti-infectious property. This reinforces the immunomodulatory role of IL-24 and represents the utility of this curated pathway map to elucidate disease-gene association and its expression changes for further functional analysis.

Further, to complement the signaling pathway, we performed a pathway enrichment analysis with the differentially expressed proteins identified from the publicly available proteomic dataset of IL-24-treated cervical cancer cells (113). The analysis revealed significantly enriched pathways, including apoptosis, immunomodulatory pathways, and cell cycle regulations (**Supplementary Figure S1**). These enriched pathways align with the key signaling modules annotated in the pathway map of IL-24, further supporting the role of IL-24 in tumor suppression and immunoregulation. This integration demonstrates the utility of the application of this signaling pathway map in gene set enrichment analysis pipelines, offering a valuable resource for future studies in the analysis of high-throughput data.

## 4 Conclusions

A majority of studies have focused on the role of IL-24 in the pathophysiology of diseases, as this multi-functional cytokine serves as both a tumor suppressant and an agent with pro- and anti-inflammatory properties. IL-24 is very well known for its tumor-suppressive activity and is utilized as a therapeutic agent in cytokine therapy against a broad spectrum of cancers. The signaling pathway map of IL-24 provides a detailed illustration of its downstream molecular targets and their signaling mechanisms. Hence, this pathway map would accelerate clinical applications of IL-24 as a therapeutic target for various disorders, to its potential as a prominent therapeutic agent in cancer, and to reshape the tumor microenvironment.

The signaling pathway map developed in this study provides a framework to visualize the molecules and their reactions reported to be induced by IL-24 in various disease conditions. This comprehensive signaling pathway can help identify gaps in signaling events and investigate further molecular reactions, including dynamic protein complexes associated with IL-24 signaling across various pathophysiological conditions. In the current scenario, the involvement of IL-24 and its signaling components in various clinical/experimental global omics datasets is not ensured through gene set enrichment analysis approaches. This is primarily due to the lack of availability of its pathway model in the existing enrichment tools. Further enrichment of datasets derived from high-throughput approaches such as single-cell transcriptomics and proteomics may provide deeper insights into the role of IL-24 in health and disease, leading to an expansion of the pathway.

To enable its utility, the IL-24 map/model is made freely available in.gpml format for their conversion into other international standard exchange formats such as BioPAX, PSI-MI, and KEGGML for their incorporation into different enrichment analysis tools. To acquire its full potential, IL-24-mediated effects in diverse preclinical models should be validated for their efficacy across different biological conditions or engaged in studies toward patient stratification based on response to IL-24-targeted interventions. The combinatorial effects of IL-24 with other cytokines or their regulation under various disease conditions also demand further investigation. This model can be customized or enhanced by the users to engage them in the analysis of omics datasets by individual researchers. The IL-24 pathway model will be made available through the web portal (https://ciods.in/il24/) to keep the pathway updated and provide it to the users.

## Data Availability

The original contributions presented in the study are included in the article/**Supplementary Material**. Further inquiries can be directed to the corresponding authors.

## Supplementary material

Supplementary Figure 1 Pathway enrichment analysis of the differentially expressed proteins from IL-24-treated cancer cells. The diagram represents the enriched pathways and their associated proteins from the differentially expressed proteins identified from a publicly available dataset of IL-24-treated cervical cancer cells.

Supplementary Data Sheet 1 A compendium of IL-24-mediated signaling events. The table provides the signaling events, the molecular events including activation/inhibition events, gene/protein regulations, enzyme/substrate reactions, protein-protein interaction, translocation events, and associated functional outcomes, together with the disease condition and experimental details. The data also provides the signaling events linked with the various pharmacological modulations of IL-24.

## References

[1] Caudell EG Mumm JB Poindexter N Ekmekcioglu S Mhashilkar AM Yang XH. The protein product of the tumor suppressor gene, melanoma differentiation-associated gene 7, exhibits immunostimulatory activity and is designated IL-24. J Immunol. (2002) 168:6041–6. doi: 10.4049/jimmunol.168.12.6041 12055212

[2] Fisher PB. Is mda-7/IL-24 a “magic bullet” for cancer? Cancer Res. (2005) 65:10128–38. doi: 10.1158/0008-5472.CAN-05-3127 16287994

[3] Zhong Y Zhang X Chong W. Interleukin-24 immunobiology and its roles in inflammatory diseases. Int J Mol Sci. (2022) 23:627. doi: 10.3390/ijms23020627 35054813 PMC8776082

[4] Jiang H Lin JJ Su ZZ Goldstein NI Fisher PB. Subtraction hybridization identifies a novel melanoma differentiation associated gene, mda-7, modulated during human melanoma differentiation, growth and progression. Oncogene. (1995) 11:2477–86. 8545104

[5] Dumoutier L Leemans C Lejeune D Kotenko SV Renauld JC. Cutting edge: STAT activation by IL-19, IL-20 and mda-7 through IL-20 receptor complexes of two types. J Immunol. (2001) 167:3545–9. doi: 10.4049/jimmunol.167.7.3545 11564763

[6] Wang M Tan Z Zhang R Kotenko SV Liang P. Interleukin 24 (MDA-7/MOB-5) signals through two heterodimeric receptors, IL-22R1/IL-20R2 and IL-20R1/IL-20R2. J Biol Chem. (2002) 277:7341–7. doi: 10.1074/jbc.M106043200 11706020

[7] Parrish-Novak J Xu W Brender T Yao L Jones C West J. Interleukins 19, 20, and 24 signal through two distinct receptor complexes. Differences in receptor-ligand interactions mediate unique biological functions. J Biol Chem. (2002) 277:47517–23. doi: 10.1074/jbc.M205114200 12351624

[8] Pestka S Krause CD Sarkar D Walter MR Shi Y Fisher PB. Interleukin-10 and related cytokines and receptors. Annu Rev Immunol. (2004) 22:929–79. doi: 10.1146/annurev.immunol.22.012703.104622 15032600

[9] Wang M Liang P. Interleukin-24 and its receptors. Immunology. (2005) 114:166–70. doi: 10.1111/j.1365-2567.2005.02094.x PMC1782067 15667561

[10] Menezes ME Bhoopathi P Pradhan AK Emdad L Das SK Guo C. Role of MDA-7/IL-24 a multifunction protein in human diseases. Adv Cancer Res. (2018) 138:143–82. doi: 10.1016/bs.acr.2018.02.005 PMC6218935 29551126

[11] Modi J Roy A Pradhan AK Kumar A Talukdar S Bhoopathi P. Insights into the mechanisms of action of MDA-7/IL-24: A ubiquitous cancer-suppressing protein. Int J Mol Sci. (2021) 23:72. doi: 10.3390/ijms23010072 35008495 PMC8744595

[12] Huang EY Madireddi MT Gopalkrishnan RV Leszczyniecka M Su Z Lebedeva IV. Genomic structure, chromosomal localization and expression profile of a novel melanoma differentiation associated (mda-7) gene with cancer specific growth suppressing and apoptosis inducing properties. Oncogene. (2001) 20:7051–63. doi: 10.1038/sj.onc.1204897 11704829

[13] Sarkar D Su ZZ Lebedeva IV Sauane M Gopalkrishnan RV Dent P. mda-7 (IL-24): signaling and functional roles. Biotechniques. (2002) Suppl:30–9. doi: 10.2144/Oct0204 12395925

[14] Smith S Lopez S Kim A Kasteri J Olumuyide E Punu K. Interleukin 24: signal transduction pathways. Cancers (Basel). (2023) 15:3365. doi: 10.3390/cancers15133365 37444474 PMC10340555

[15] Logsdon NJ Deshpande A Harris BD Rajashankar KR Walter MR. Structural basis for receptor sharing and activation by interleukin-20 receptor-2 (IL-20R2) binding cytokines. Proc Natl Acad Sci U S A. (2012) 109:12704–9. doi: 10.1073/pnas.1117551109 PMC3412030 22802649

[16] Sieger KA Mhashilkar AM Stewart A Sutton RB Strube RW Chen SY. The tumor suppressor activity of MDA-7/IL-24 is mediated by intracellular protein expression in NSCLC cells. Mol Ther. (2004) 9:355–67. doi: 10.1016/j.ymthe.2003.11.014 15006602

[17] Sauane M Lebedeva IV Su ZZ Choo HT Randolph A Valerie K. Melanoma differentiation associated gene-7/interleukin-24 promotes tumor cell-specific apoptosis through both secretory and nonsecretory pathways. Cancer Res. (2004) 64:2988–93. doi: 10.1158/0008-5472.CAN-04-0200 15126330

[18] Menezes ME Bhatia S Bhoopathi P Das SK Emdad L Dasgupta S. MDA-7/IL-24: multifunctional cancer killing cytokine. Adv Exp Med Biol. (2014) 818:127–53. doi: 10.1007/978-1-4471-6458-6_6 PMC4633013 25001534

[19] Gupta P Su ZZ Lebedeva IV Sarkar D Sauane M Emdad L. mda-7/IL-24: multifunctional cancer-specific apoptosis-inducing cytokine. Pharmacol Ther. (2006) 111:596–628. doi: 10.1016/j.pharmthera.2005.11.005 16464504 PMC1781515

[20] Ma Y Chen H Wang Q Luo F Yan J Zhang XL. IL-24 protects against Salmonella typhimurium infection by stimulating early neutrophil Th1 cytokine production, which in turn activates CD8+ T cells. Eur J Immunol. (2009) 39:3357–68. doi: 10.1002/eji.200939678 19830736

[21] Gopalkrishnan RV Sauane M Fisher PB. Cytokine and tumor cell apoptosis inducing activity of mda-7/IL-24. Int Immunopharmacol. (2004) 4:635–47. doi: 10.1016/j.intimp.2004.01.015 15120649

[22] Zhang Y Liu Y Xu Y. Interleukin-24 regulates T cell activity in patients with colorectal adenocarcinoma. Front Oncol. (2019) 9:1401. doi: 10.3389/fonc.2019.01401 31921658 PMC6915036

[23] Anuradha R George PJ Hanna LE Kumaran P Chandrasekaran V Nutman TB. Expansion of parasite-specific CD4+ and CD8+ T cells expressing IL-10 superfamily cytokine members and their regulation in human lymphatic filariasis. PloS Negl Trop Dis. (2014) 8:e2762. doi: 10.1371/journal.pntd.0002762 24699268 PMC3974669

[24] Persaud L De Jesus D Brannigan O Richiez-Paredes M Huaman J Alvarado G. Mechanism of action and applications of interleukin 24 in immunotherapy. Int J Mol Sci. (2016) 17:869. doi: 10.3390/ijms17060869 27271601 PMC4926403

[25] Zhang K Hu W Li F Wen C Zhou L Zhang L. IL-24 improves efficacy of CAR-T cell therapy by targeting stemness of tumor cells. Br J Cancer. (2024) 130:1337–47. doi: 10.1038/s41416-024-02601-1 PMC11015030 38347092

[26] Hu Q Zhang Y Wang P Zhou M Hu Z Liu C. IL-24 armored CAR19-T cells show enhanced antitumor activity and persistence. Signal Transduct Target Ther. (2021) 6:14. doi: 10.1038/s41392-020-00380-8 33441538 PMC7806903

[27] Liu Z Guo C Das SK Yu X Pradhan AK Li X. Engineering T cells to express tumoricidal MDA-7/IL24 enhances cancer immunotherapy. Cancer Res. (2021) 81:2429–41. doi: 10.1158/0008-5472.CAN-20-2604 PMC8362838 33727225

[28] Ramesh R Ioannides CG Roth JA Chada S. Adenovirus-mediated interleukin (IL)-24 immunotherapy for cancer. Methods Mol Biol. (2010) 651:241–70. doi: 10.1007/978-1-60761-786-0_14 20686970

[29] Dent P Yacoub A Hamed HA Park MA Dash R Bhutia SK. The development of MDA-7/IL-24 as a cancer therapeutic. Pharmacol Ther. (2010) 128:375–84. doi: 10.1016/j.pharmthera.2010.08.001 PMC2947573 20732354

[30] Fisher PB Gopalkrishnan RV Chada S Ramesh R Grimm EA Rosenfeld MR. mda-7/IL-24, a novel cancer selective apoptosis inducing cytokine gene: from the laboratory into the clinic. Cancer Biol Ther. (2003) 2:S23–37. doi: 10.4161/cbt.458 14508078

[31] Lebedeva IV Sauane M Gopalkrishnan RV Sarkar D Su ZZ Gupta P. mda-7/IL-24: exploiting cancer’s Achilles’ heel. Mol Ther. (2005) 11:4–18. doi: 10.1016/j.ymthe.2004.08.012 15585401

[32] Cunningham CC Chada S Merritt JA Tong A Senzer N Zhang Y. Clinical and local biological effects of an intratumoral injection of mda-7 (IL24; INGN 241) in patients with advanced carcinoma: a phase I study. Mol Ther. (2005) 11:149–59. doi: 10.1016/j.ymthe.2004.09.019 15585416

[33] Tong AW Nemunaitis J Su D Zhang Y Cunningham C Senzer N. Intratumoral injection of INGN 241, a nonreplicating adenovector expressing the melanoma-differentiation associated gene-7 (mda-7/IL24): biologic outcome in advanced cancer patients. Mol Ther. (2005) 11:160–72. doi: 10.1016/j.ymthe.2004.09.021 15585417

[34] Lebedeva IV Emdad L Su ZZ Gupta P Sauane M Sarkar D. mda-7/IL-24, novel anticancer cytokine: focus on bystander antitumor, radiosensitization and antiangiogenic properties and overview of the phase I clinical experience (Review). Int J Oncol. (2007) 31:985–1007. doi: 10.3892/ijo.31.5.985 17912425

[35] Inoue S Shanker M Miyahara R Gopalan B Patel S Oida Y. MDA-7/IL-24-based cancer gene therapy: translation from the laboratory to the clinic. Curr Gene Ther. (2006) 6:73–91. doi: 10.2174/156652306775515574 16475947

[36] Fisher PB Sarkar D Lebedeva IV Emdad L Gupta P Sauane M. Melanoma differentiation associated gene-7/interleukin-24 (mda-7/IL-24): novel gene therapeutic for metastatic melanoma. Toxicol Appl Pharmacol. (2007) 224:300–7. doi: 10.1016/j.taap.2006.11.021 PMC2739016 17208263

[37] Aravind A Palollathil A Rex DAB Kumar KMK Vijayakumar M Shetty R. A multi-cellular molecular signaling and functional network map of C-C motif chemokine ligand 18 (CCL18): a chemokine with immunosuppressive and pro-tumor functions. J Cell Commun Signal. (2022) 16:293–300. doi: 10.1007/s12079-021-00633-3 34196939 PMC8891403

[38] Balakrishnan L Soman S Patil YB Advani J Thomas JK Desai DV. IL-11/IL11RA receptor mediated signaling: a web accessible knowledgebase. Cell Commun Adhes. (2013) 20:81–6. doi: 10.3109/15419061.2013.791683 23631681

[39] Sharma J Balakrishnan L Datta KK Sahasrabuddhe NA Khan AA Sahu A. A knowledgebase resource for interleukin-17 family mediated signaling. J Cell Commun Signal. (2015) 9:291–6. doi: 10.1007/s12079-015-0297-3 PMC4580681 26077014

[40] Raju R Gadakh S Gopal P George B Advani J Soman S. Differential ligand-signaling network of CCL19/CCL21-CCR7 system. Database (Oxford). (2015) 2015:bav106. doi: 10.1093/database/bav106 26504105 PMC4620938

[41] Shaji V Dagamajalu S Sanjeev D George M Kanekar S Prasad G. Deciphering the receptor-mediated signaling pathways of interleukin-19 and interleukin-20. J Interferon Cytokine Res. (2024) 44(9):388–98. 10.1089/jir.2024.0009 38451706

[42] Kandasamy K Keerthikumar S Raju R Keshava Prasad TS Ramachandra YL Mohan S. PathBuilder–open source software for annotating and developing pathway resources. Bioinformatics. (2009) 25:2860–2. doi: 10.1093/bioinformatics/btp453 PMC2781757 19628504

[43] Kandasamy K Mohan SS Raju R Keerthikumar S Kumar GS Venugopal AK. NetPath: a public resource of curated signal transduction pathways. Genome Biol. (2010) 11:R3. doi: 10.1186/gb-2010-11-1-r3 20067622 PMC2847715

[44] Kutmon M van Iersel MP Bohler A Kelder T Nunes N Pico AR. PathVisio 3: an extendable pathway analysis toolbox. PloS Comput Biol. (2015) 11:e1004085. doi: 10.1371/journal.pcbi.1004085 25706687 PMC4338111

[45] Szklarczyk D Kirsch R Koutrouli M Nastou K Mehryary F Hachilif R. The STRING database in 2023: protein-protein association networks and functional enrichment analyses for any sequenced genome of interest. Nucleic Acids Res. (2023) 51:D638–D46. doi: 10.1093/nar/gkac1000 PMC9825434 36370105

[46] Shannon P Markiel A Ozier O Baliga NS Wang JT Ramage D. Cytoscape: a software environment for integrated models of biomolecular interaction networks. Genome Res. (2003) 13:2498–504. doi: 10.1101/gr.1239303 PMC403769 14597658

[47] Bader GD Hogue CW. An automated method for finding molecular complexes in large protein interaction networks. BMC Bioinf. (2003) 4:2. doi: 10.1186/1471-2105-4-2 PMC149346 12525261

[48] Milacic M Beavers D Conley P Gong C Gillespie M Griss J. The reactome pathway knowledgebase 2024. Nucleic Acids Res. (2024) 52:D672–D8. doi: 10.1093/nar/gkad1025 PMC10767911 37941124

[49] Trivella DB Ferreira-Junior JR Dumoutier L Renauld JC Polikarpov I. Structure and function of interleukin-22 and other members of the interleukin-10 family. Cell Mol Life Sci. (2010) 67:2909–35. doi: 10.1007/s00018-010-0380-0 PMC11115847 20454917

[50] Dash R Bhutia SK Azab B Su ZZ Quinn BA Kegelmen TP. mda-7/IL-24: a unique member of the IL-10 gene family promoting cancer-targeted toxicity. Cytokine Growth Factor Rev. (2010) 21:381–91. doi: 10.1016/j.cytogfr.2010.08.004 PMC3164830 20926331

[51] Do W Herrera C Mighty J Shumskaya M Redenti SM Sauane M. Sigma 1 Receptor plays a prominent role in IL-24-induced cancer-specific apoptosis. Biochem Biophys Res Commun. (2013) 439:215–20. doi: 10.1016/j.bbrc.2013.08.057 23988449

[52] Persaud L Mighty J Zhong X Francis A Mendez M Muharam H. IL-24 Promotes Apoptosis through cAMP-Dependent PKA Pathways in Human Breast Cancer Cells. Int J Mol Sci. (2018) 19:3561. doi: 10.3390/ijms19113561 30424508 PMC6274865

[53] Oida Y Gopalan B Miyahara R Branch CD Chiao P Chada S. Inhibition of nuclear factor-kappaB augments antitumor activity of adenovirus-mediated melanoma differentiation-associated gene-7 against lung cancer cells via mitogen-activated protein kinase kinase kinase 1 activation. Mol Cancer Ther. (2007) 6:1440–9. doi: 10.1158/1535-7163.MCT-06-0374 17431123

[54] Wang L Feng Z Wu H Zhang S Pu Y Bian H. Melanoma differentiation-associated gene-7/interleukin-24 as a potential prognostic biomarker and second primary Malignancy indicator in head and neck squamous cell carcinoma patients. Tumour Biol. (2014) 35:10977–85. doi: 10.1007/s13277-014-2392-0 25091574

[55] Tian H Zhang D Gao Z Li H Zhang B Zhang Q. MDA-7/IL-24 inhibits Nrf2-mediated antioxidant response through activation of p38 pathway and inhibition of ERK pathway involved in cancer cell apoptosis. Cancer Gene Ther. (2014) 21:416–26. doi: 10.1038/cgt.2014.45 25236495

[56] Gopalan B Litvak A Sharma S Mhashilkar AM Chada S Ramesh R. Activation of the Fas-FasL signaling pathway by MDA-7/IL-24 kills human ovarian cancer cells. Cancer Res. (2005) 65:3017–24. doi: 10.1158/0008-5472.CAN-04-3758 15833826

[57] Tian H Zhang DF Zhang BF Li HZ Zhang Q Li LT. Melanoma differentiation associated gene-7/interleukin-24 induces caspase-3 denitrosylation to facilitate the activation of cancer cell apoptosis. J Interferon Cytokine Res. (2015) 35:157–67. doi: 10.1089/jir.2014.0061 25347351

[58] Pataer A Chada S Hunt KK Roth JA Swisher SG. Adenoviral melanoma differentiation-associated gene 7 induces apoptosis in lung cancer cells through mitochondrial permeability transition-independent cytochrome c release. J Thorac Cardiovasc Surg. (2003) 125:1328–35. doi: 10.1016/S0022-5223(02)73247-9 12830052

[59] Valiyari S Salami M Mahdian R Shokrgozar MA Oloomi M Mohammadi Farsani A. sIL-24 peptide, a human interleukin-24 isoform, induces mitochondrial-mediated apoptosis in human cancer cells. Cancer Chemother Pharmacol. (2017) 80:451–9. doi: 10.1007/s00280-017-3370-1 28653252

[60] Zerbini LF Czibere A Wang Y Correa RG Otu H Joseph M. A novel pathway involving melanoma differentiation associated gene-7/interleukin-24 mediates nonsteroidal anti-inflammatory drug-induced apoptosis and growth arrest of cancer cells. Cancer Res. (2006) 66:11922–31. doi: 10.1158/0008-5472.CAN-06-2068 17178890

[61] Pradhan AK Bhoopathi P Talukdar S Scheunemann D Sarkar D Cavenee WK. MDA-7/IL-24 regulates the miRNA processing enzyme DICER through downregulation of MITF. Proc Natl Acad Sci U S A. (2019) 116:5687–92. doi: 10.1073/pnas.1819869116 PMC6431152 30842276

[62] Rastegari M Shiri A Behzad-Behbahani A Rasoolian M Zare F Rafiei G. The evaluation of tLyP-1-bound mda-7/IL-24 killing activity on a liver tumor cell line. Cancer Biother Radiopharm. (2021) 36:827–36. doi: 10.1089/cbr.2019.3080 32493109

[63] Pataer A Hu W Xiaolin L Chada S Roth JA Hunt KK. Adenoviral endoplasmic reticulum-targeted mda-7/interleukin-24 vector enhances human cancer cell killing. Mol Cancer Ther. (2008) 7:2528–35. doi: 10.1158/1535-7163.MCT-08-0083 PMC2597048 18723497

[64] Pradhan AK Talukdar S Bhoopathi P Shen XN Emdad L Das SK. mda-7/IL-24 Mediates Cancer Cell-Specific Death via Regulation of miR-221 and the Beclin-1 Axis. Cancer Res. (2017) 77:949–59. doi: 10.1158/0008-5472.CAN-16-1731 PMC5313338 27940575

[65] Bhutia SK Das SK Azab B Dash R Su ZZ Lee SG. Autophagy switches to apoptosis in prostate cancer cells infected with melanoma differentiation associated gene-7/interleukin-24 (mda-7/IL-24). Autophagy. (2011) 7:1076–7. doi: 10.4161/auto.7.9.16163 PMC3210317 21610321

[66] Saeki T Mhashilkar A Chada S Branch C Roth JA Ramesh R. Tumor-suppressive effects by adenovirus-mediated mda-7 gene transfer in non-small cell lung cancer cell *in vitro* . Gene Ther. (2000) 7:2051–7. doi: 10.1038/sj.gt.3301330 11175318

[67] Inoue S Branch CD Gallick GE Chada S Ramesh R. Inhibition of Src kinase activity by Ad-mda7 suppresses vascular endothelial growth factor expression in prostate carcinoma cells. Mol Ther. (2005) 12:707–15. doi: 10.1016/j.ymthe.2005.05.015 16054437

[68] Saito Y Miyahara R Gopalan B Litvak A Inoue S Shanker M. Selective induction of cell cycle arrest and apoptosis in human prostate cancer cells through adenoviral transfer of the melanoma differentiation-associated -7 (mda-7)/interleukin-24 (IL-24) gene. Cancer Gene Ther. (2005) 12:238–47. doi: 10.1038/sj.cgt.7700780 15578066

[69] Zhuo B Shi Y Qin H Sun Q Li Z Zhang F. Interleukin-24 inhibits osteosarcoma cell migration and invasion via the JNK/c-Jun signaling pathways. Oncol Lett. (2017) 13:4505–11. doi: 10.3892/ol.2017.5990 PMC5453038 28599451

[70] Huo W Li ZM Zhu XM Bao YM An LJ. MDA-7/IL-24 suppresses tumor adhesion and invasive potential in hepatocellular carcinoma cell lines. Oncol Rep. (2013) 30:986–92. doi: 10.3892/or.2013.2507 23722307

[71] Hamed HA Yacoub A Park MA Eulitt PJ Dash R Sarkar D. Inhibition of multiple protective signaling pathways and Ad.5/3 delivery enhances mda-7/IL-24 therapy of Malignant glioma. Mol Ther. (2010) 18:1130–42. doi: 10.1038/mt.2010.29 PMC2889737 20179672

[72] Li QY Shi Y Huang DH Yang T Wang JH Yan GH. Cytokine-induced killer cells combined with dendritic cells inhibited liver cancer cells. Int J Clin Exp Med. (2015) 8:5601–10. PMC4483965 26131143

[73] Lin T Wang D Chen J Zhang Z Zhao Y Wu Z. IL-24 inhibits the Malignancy of human glioblastoma cells via destabilization of Zeb1. Biol Chem. (2021) 402:839–48. doi: 10.1515/hsz-2020-0373 33894112

[74] Bhutia SK Das SK Azab B Menezes ME Dent P Wang XY. Targeting breast cancer-initiating/stem cells with melanoma differentiation-associated gene-7/interleukin-24. Int J Cancer. (2013) 133:2726–36. doi: 10.1002/ijc.28289 PMC4334374 23720015

[75] Tian H Wang J Zhang B Di J Chen F Li H. MDA-7/IL-24 induces Bcl-2 denitrosylation and ubiquitin-degradation involved in cancer cell apoptosis. PloS One. (2012) 7:e37200. doi: 10.1371/journal.pone.0037200 22629368 PMC3357419

[76] Zhuo B Wang X Shen Y Li J Li S Li Y. Interleukin-24 inhibits the phenotype and tumorigenicity of cancer stem cell in osteosarcoma via downregulation Notch and Wnt/beta-catenin signaling. J Bone Oncol. (2021) 31:100403. doi: 10.1016/j.jbo.2021.100403 34804789 PMC8581362

[77] Emdad L Lebedeva IV Su ZZ Sarkar D Dent P Curiel DT. Melanoma differentiation associated gene-7/interleukin-24 reverses multidrug resistance in human colorectal cancer cells. Mol Cancer Ther. (2007) 6:2985–94. doi: 10.1158/1535-7163.MCT-07-0399 18025283

[78] Fang P Zhang X Gao Y Ding CR Cui F Jiao SC. Reversal effect of melanoma differentiation associated gene-7/interleukin-24 on multidrug resistance in human hepatocellular carcinoma cells. Anat Rec (Hoboken). (2012) 295:1639–46. doi: 10.1002/ar.22551 22899557

[79] Panneerselvam J Jin J Shanker M Lauderdale J Bates J Wang Q. IL-24 inhibits lung cancer cell migration and invasion by disrupting the SDF-1/CXCR4 signaling axis. PloS One. (2015) 10:e0122439. doi: 10.1371/journal.pone.0122439 25775124 PMC4361489

[80] Deng WG Kwon J Ekmekcioglu S Poindexter NJ Grimm EA. IL-24 gene transfer sensitizes melanoma cells to erlotinib through modulation of the Apaf-1 and Akt signaling pathways. Melanoma Res. (2011) 21:44–56. doi: 10.1097/CMR.0b013e3283382155 20216471 PMC2945428

[81] Liu J Zhang Y Sun P Xie Y Xiang J Yang J. Enhanced therapeutic efficacy of adenovirus-mediated interleukin-24 gene therapy combined with ionizing radiotherapy for nasopharyngeal carcinoma. Oncol Rep. (2013) 30:1165–74. doi: 10.3892/or.2013.2550 23783436

[82] Pal I Sarkar S Rajput S Dey KK Chakraborty S Dash R. BI-69A11 enhances susceptibility of colon cancer cells to mda-7/IL-24-induced growth inhibition by targeting Akt. Br J Cancer. (2014) 111:101–11. doi: 10.1038/bjc.2014.227 PMC4090725 24892445

[83] Shanker M Gopalan B Patel S Bocangel D Chada S Ramesh R. Vitamin E succinate in combination with mda-7 results in enhanced human ovarian tumor cell killing through modulation of extrinsic and intrinsic apoptotic pathways. Cancer Lett. (2007) 254:217–26. doi: 10.1016/j.canlet.2007.03.004 17449172

[84] Hamed HA Yacoub A Park MA Eulitt P Sarkar D Dimitrie IP. OSU-03012 enhances Ad.7-induced GBM cell killing via ER stress and autophagy and by decreasing expression of mitochondrial protective proteins. Cancer Biol Ther. (2010) 9:526–36. doi: 10.4161/cbt.9.7.11116 PMC2888700 20107314

[85] Seong RK Choi YK Shin OS. MDA7/IL-24 is an anti-viral factor that inhibits influenza virus replication. J Microbiol. (2016) 54:695–700. doi: 10.1007/s12275-016-6383-2 27687232

[86] Onody A Veres-Szekely A Pap D Rokonay R Szebeni B Sziksz E. Interleukin-24 regulates mucosal remodeling in inflammatory bowel diseases. J Transl Med. (2021) 19:237. doi: 10.1186/s12967-021-02890-7 34078403 PMC8173892

[87] Kumari S Bonnet MC Ulvmar MH Wolk K Karagianni N Witte E. Tumor necrosis factor receptor signaling in keratinocytes triggers interleukin-24-dependent psoriasis-like skin inflammation in mice. Immunity. (2013) 39:899–911. doi: 10.1016/j.immuni.2013.10.009 24211183

[88] Shao J Zhang B Yu JJ Wei CY Zhou WJ Chang KK. Macrophages promote the growth and invasion of endometrial stromal cells by downregulating IL-24 in endometriosis. Reproduction. (2016) 152:673–82. doi: 10.1530/REP-16-0278 27624484

[89] Feng KN Meng P Zhang M Zou XL Li S Huang CQ. IL-24 contributes to neutrophilic asthma in an IL-17A-dependent manner and is suppressed by IL-37. Allergy Asthma Immunol Res. (2022) 14:505–27. doi: 10.4168/aair.2022.14.5.505 PMC9523421 36174993

[90] Feng KN Meng P Zou XL Zhang M Li HK Yang HL. IL-37 protects against airway remodeling by reversing bronchial epithelial-mesenchymal transition via IL-24 signaling pathway in chronic asthma. Respir Res. (2022) 23:244. doi: 10.1186/s12931-022-02167-7 36100847 PMC9472332

[91] Mitamura Y Nunomura S Furue M Izuhara K. IL-24: A new player in the pathogenesis of pro-inflammatory and allergic skin diseases. Allergol Int. (2020) 69:405–11. doi: 10.1016/j.alit.2019.12.003 31980374

[92] Wu B Huang C Kato-Maeda M Hopewell PC Daley CL Krensky AM. IL-24 modulates IFN-gamma expression in patients with tuberculosis. Immunol Lett. (2008) 117:57–62. doi: 10.1016/j.imlet.2007.11.018 18199488 PMC2679252

[93] Rokonay R Veres-Szekely A Szebeni B Pap D Lippai R Beres NJ. Role of IL-24 in the mucosal remodeling of children with coeliac disease. J Transl Med. (2020) 18:36. doi: 10.1186/s12967-020-02221-2 31973719 PMC6977354

[94] Andoh A Shioya M Nishida A Bamba S Tsujikawa T Kim-Mitsuyama S. Expression of IL-24, an activator of the JAK1/STAT3/SOCS3 cascade, is enhanced in inflammatory bowel disease. J Immunol. (2009) 183:687–95. doi: 10.4049/jimmunol.0804169 19535621

[95] Lee KM Kang HA Park M Lee HY Choi HR Yun CH. Interleukin-24 attenuates beta-glycerophosphate-induced calcification of vascular smooth muscle cells by inhibiting apoptosis, the expression of calcification and osteoblastic markers, and the Wnt/beta-catenin pathway. Biochem Biophys Res Commun. (2012) 428:50–5. doi: 10.1016/j.bbrc.2012.09.145 23063979

[96] Wang Z Wang Y Chen Y Lv J. The IL-24 gene protects human umbilical vein endothelial cells against H(2)O(2)-induced injury and may be useful as a treatment for cardiovascular disease. Int J Mol Med. (2016) 37:581–92. doi: 10.3892/ijmm.2016.2466 PMC4771102 26820392

[97] Wang HH Huang JH Sue MH Ho WC Hsu YH Chang KC. Interleukin-24 protects against liver injury in mouse models. EBioMedicine. (2021) 64:103213. doi: 10.1016/j.ebiom.2021.103213 33508745 PMC7841303

[98] Wang J Hu B Zhao Z Zhang H Zhang H Zhao Z. Intracellular XBP1-IL-24 axis dismantles cytotoxic unfolded protein response in the liver. Cell Death Dis. (2020) 11:17. doi: 10.1038/s41419-019-2209-6 31907348 PMC6944701

[99] Zhang X Hu C Zhong Y Qiao D Chi W Shen H. Multifunctional interleukin-24 resolves neuroretina autoimmunity via diverse mechanisms. Int J Mol Sci. (2022) 23:11988. doi: 10.3390/ijms231911988 36233291 PMC9570500

[100] Sie C Kant R Peter C Muschaweckh A Pfaller M Nirschl L. IL-24 intrinsically regulates Th17 cell pathogenicity in mice. J Exp Med. (2022) 219:e20212443. doi: 10.1084/jem.20212443 35819408 PMC9280194

[101] Chong WP Mattapallil MJ Raychaudhuri K Bing SJ Wu S Zhong Y. The cytokine IL-17A limits th17 pathogenicity via a negative feedback loop driven by autocrine induction of IL-24. Immunity. (2020) 53:384–97 e5. doi: 10.1016/j.immuni.2020.06.022 32673565 PMC7362799

[102] Ma YF Ren Y Wu CJ Zhao XH Xu H Wu DZ. Interleukin (IL)-24 transforms the tumor microenvironment and induces anticancer immunity in a murine model of colon cancer. Mol Immunol. (2016) 75:11–20. doi: 10.1016/j.molimm.2016.05.010 27209087

[103] Yu X Miao J Xia W Gu ZJ. Immunogenicity moderation effect of interleukin-24 on myelogenous leukemia cells. Anticancer Drugs. (2018) 29:353–63. doi: 10.1097/CAD.0000000000000606 29420334

[104] Miyahara R Banerjee S Kawano K Efferson C Tsuda N Miyahara Y. Melanoma differentiation-associated gene-7 (mda-7)/interleukin (IL)-24 induces anticancer immunity in a syngeneic murine model. Cancer Gene Ther. (2006) 13:753–61. doi: 10.1038/sj.cgt.7700954 16543916

[105] Yan S Zhang H Xie Y Sheng W Xiang J Ye Z. Recombinant human interleukin-24 suppresses gastric carcinoma cell growth *in vitro* and *in vivo* . Cancer Invest. (2010) 28:85–93. doi: 10.3109/07357900903095672 19916746

[106] Ma Y Chen HD Wang Y Wang Q Li Y Zhao Y. Interleukin 24 as a novel potential cytokine immunotherapy for the treatment of Mycobacterium tuberculosis infection. Microbes Infect. (2011) 13:1099–110. doi: 10.1016/j.micinf.2011.06.012 21787878

[107] Moreno Ayala MA Gottardo MF Imsen M Asad AS Bal de Kier Joffe E Casares N. Therapeutic blockade of Foxp3 in experimental breast cancer models. Breast Cancer Res Treat. (2017) 166:393–405. doi: 10.1007/s10549-017-4414-2 28756536

[108] Saleh R Elkord E. FoxP3(+) T regulatory cells in cancer: Prognostic biomarkers and therapeutic targets. Cancer Lett. (2020) 490:174–85. doi: 10.1016/j.canlet.2020.07.022 32721551

[109] Abhinand CS Raju R Soumya SJ Arya PS Sudhakaran PR. VEGF-A/VEGFR2 signaling network in endothelial cells relevant to angiogenesis. J Cell Commun Signal. (2016) 10:347–54. doi: 10.1007/s12079-016-0352-8 PMC5143324 27619687

[110] Cvetkovic J Jacobi RHJ Miranda-Bedate A Pham N Kutmon M Groot J. Human monocytes exposed to SARS-coV-2 display features of innate immune memory producing high levels of CXCL10 upon restimulation. J Innate Immun. (2023) 15:911–24. doi: 10.1159/000535120 PMC10718582 37989107

[111] Marotel M Villard M Drouillard A Tout I Besson L Allatif O. Peripheral natural killer cells in chronic hepatitis B patients display multiple molecular features of T cell exhaustion. Elife. (2021) 10:e60095. doi: 10.7554/eLife.60095.sa2 33507150 PMC7870135

[112] Pontrelli P Rascio F Zaza G Accetturo M Simone S Infante B. Interleukin-27 is a potential marker for the onset of post-transplant Malignancies. Nephrol Dial Transplant. (2019) 34:157–66. doi: 10.1093/ndt/gfy206 30059989

[113] Wei L Wang Z Cui T Yi F Bu Y Ding S. Proteomic analysis of cervical cancer cells treated with adenovirus-mediated MDA-7. Cancer Biol Ther. (2008) 7:510–6. doi: 10.4161/cbt.7.4.5478 18299662

