# Supplementary figures and images for: An assembled molecular signaling map of interleukin-24: a resource to decipher its multifunctional immunoregulatory role in pathophysiological conditions
Source: Front Immunol. 2025 Jun 30;16:1608101. doi: 10.3389/fimmu.2025.1608101 (PMC12257977; doi:10.3389/fimmu.2025.1608101)

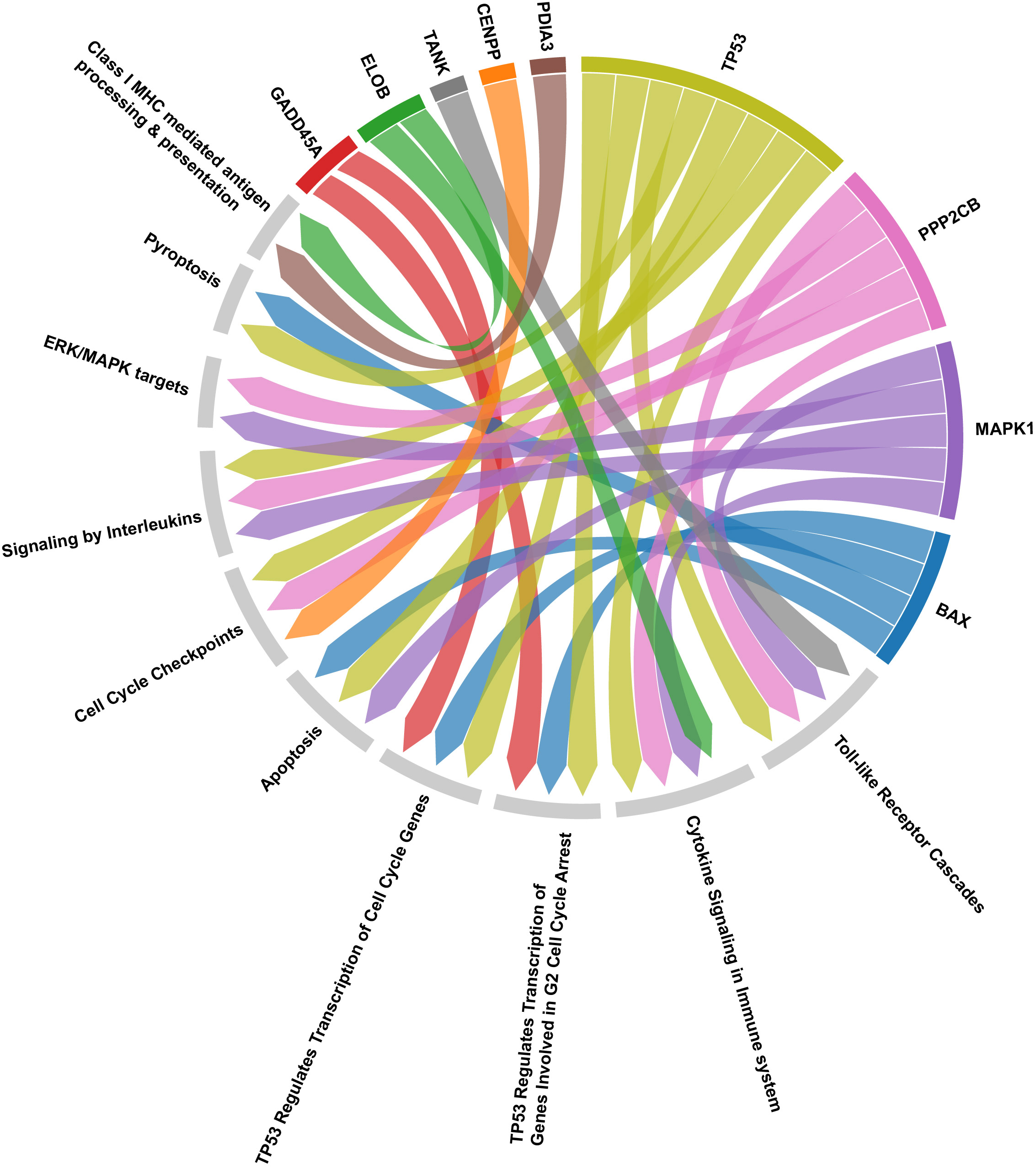

Supplement: Supplementary Figure 1 — Pathway enrichment analysis of the differentially expressed proteins from IL-24-treated cancer cells. The diagram represents the enriched pathways and their associated proteins from the differentially expressed proteins identified from a publicly available dataset of IL-24-treated cervical cancer cells. [file Image1.jpeg]
